# Association of *miR-146a*, *miR-149*, *miR-196a2*, and *miR-499* Polymorphisms with Ossification of the Posterior Longitudinal Ligament of the Cervical Spine

**DOI:** 10.1371/journal.pone.0159756

**Published:** 2016-07-25

**Authors:** Jae Joon Lim, Dong Ah Shin, Young Joo Jeon, Hemant Kumar, Seil Sohn, Hyoung Sik Min, Jang Bo Lee, Sung Uk Kuh, Keung Nyun Kim, Jung Oh Kim, Ok Joon Kim, Alexander E. Ropper, Nam Keun Kim, In Bo Han

**Affiliations:** 1 Department of Neurosurgery, CHA Bundang Medical Center, CHA University, Seongnam, Korea; 2 Department of Neurosurgery, Severance Hospital, Yonsei University, Seoul, Korea; 3 Genome Research Center, Green Cross Genome, Yongin, Korea; 4 Department of Neurosurgery, College of Medicine, Korea University, Seoul, Korea; 5 Department of Neurosurgery, Gangnam Severance Hospital, Yonsei University, Seoul, Korea; 6 Department of Biomedical Science, College of Life Science, CHA University, Seongnam, Korea; 7 Department of Neurology, CHA Bundang Medical Center, CHA University, Seongnam, Korea; 8 Department of Neurosurgery, Baylor College of Medicine, Houston, Texas, United States of America; The University of Adelaide, AUSTRALIA

## Abstract

**Background:**

Ossification of the posterior longitudinal ligament (OPLL) of the spine is considered a multifactorial and polygenic disease. We aimed to investigate the association between four single nucleotide polymorphisms (SNPs) of pre-miRNAs [*miR-146a*C>G (rs2910164), *miR-149*T>C (rs2292832), *miR-196a2*T>C (rs11614913), and *miR*-499A>G (rs3746444)] and the risk of cervical OPLL in the Korean population.

**Methods:**

The genotypic frequencies of these four SNPs were analyzed in 207 OPLL patients and 200 controls by polymerase chain reaction-restriction fragment length polymorphism (PCR-RFLP) assay.

**Findings:**

For four SNPs in pre-miRNAs, no significant differences were found between OPLL patients and controls. However, subgroup analysis based on OPLL subgroup (continuous: continuous type plus mixed type, segmental: segmental and localized type) showed that *miR-499*GG genotype was associated with an increased risk of segmental type OPLL (adjusted odds ratio = 4.314 with 95% confidence interval: 1.109–16.78). In addition, some allele combinations (C-T-T-G, G-T-T-A, and G-T-C-G of *miR-146a/-149/-196a2/-499*) and combined genotypes (*miR-149*TC*/miR-196a2*TT) were associated with increased OPLL risk, whereas the G-T-T-G and G-C-C-G allele combinations were associated with decreased OPLL risk.

**Conclusion:**

The results indicate that GG genotype of *miR*-499 is associated with significantly higher risks of OPLL in the segmental OPLL group. The *miR-146a/-149/-196a2/-499* allele combinations may be a genetic risk factor for cervical OPLL in the Korean population.

## Introduction

Ossification of the posterior longitudinal ligament (OPLL) of the spine is a common disease characterized by growth of the PLL, development of ossification centers and eventual calcification and formation of mature ectopic bone. It generally occurs in the aging population, affecting 0.8–3.0% of eastern Asians [1–3]. OPLL presents with myelopathy and/or radiculopathy due to chronic compression of the spinal cord and nerve roots. It is a multi-factorial disease influenced by genetic and environmental factors. Several lines of evidence suggest that genetic factors contribute to its etiology and pathogenesis. Affected sibling-pair linkage studies, candidate gene association studies, and a genome-wide association study identified a number of genes or loci that are linked to OPLL susceptibility [1–11]. To date, however, no genetic study has been designed to identify microRNA (miRNA) polymorphisms that may be associated with OPLL risk.

**Fig 1:**
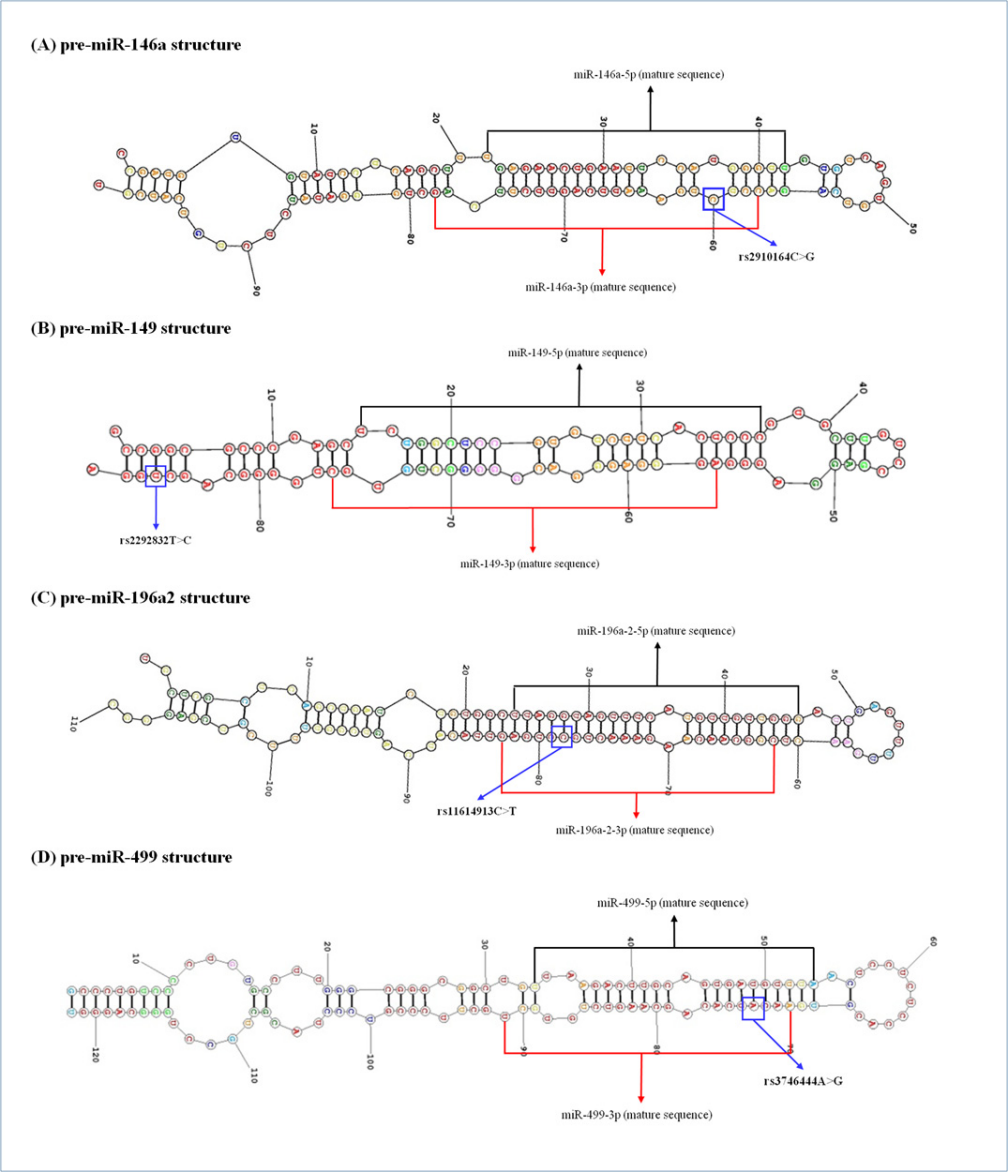
Structures and the locations of single nucleotide polymorphisms of *pre-miR-146a*, *miR-149*, *miR-196a2*, and *miR-499*.

miRNAs are small, single-stranded, non-protein-coding RNAs that pair with sites in 3′-untranslated regions (3′-UTR) of messenger RNAs (mRNAs) to downregulate their expression [12]. Recently, several studies demonstrated that single nucleotide polymorphisms (SNPs) present in miRNA genes can alter miRNA expression and/or maturation and can cause a wide variety of pathology. Several SNPs have been reported in pre-miRNA (miRNA precursor) sequences with documented effects on the miRNA expression and function. Four well-known SNPs in pre-miRNA sequences (*miR-146a* C>G [rs2910164], *miR-149* T>C [rs2292832], *miR-196a2* T>C [rs11614913], and *miR*-*499* A>G [rs3746444]) have been extensively studied in various types of cancer [13–17], ischemic stroke [18], moyamoya disease [19], premature ovarian failure [20], and spontaneous abortion [21] by our group and by others. Among these four SNPs, the *miR-149* rs2292832 SNP is the only one located outside the mature region of *pre-mir-149*. However, *miR-146a* C>G [rs2910164], *miR-196a2* T>C [rs11614913] and *miR*-*499* A>G [rs3746444]) polymorphisms occurs in the 3p strand in mature miRNA regions (Fig 1), and they may have influence on both the binding of target mRNAs to 3p mature miRNAs and pre-miRNA maturation of 5p and 3p miRNAs [14, 22–26]. In addition, the SNPs in the pre-miRNA region of *miR-146a*, *miR-196a2*, and *miR*-*499* not only influences mature miRNA expression, but also affects target gene expression [14, 22–26].

*miR-146a* has been found to be involved in the regulation of osteogenesis [27,28], inflammation [29], and human chondrocyte apoptosis [30]. It has been reported that the expression of the methylenetetrahydrofolate reductase (*MTHFR*) gene may be regulated by *miR-149* and a common polymorphism (C677T) in the gene encoding MTHFR may be associated with bone mineral density [31,32]. *miR-196a2* can target annexin A1 (ANX A1), which is related to anti-inflammation [33]. *miR-499* can influence the inflammatory reaction by modulating C-reactive protein (CRP) [34,35]. Considering that OPLL is considered to result from progressive inflammation of the ligaments [36] and an enhanced potential for osteogenesis [37], the four miRNAs targets may play a role in the pathogenesis of OPLL by modulating inflammation pathways and osteogenic differentiation. However, there is limited literature critically examining the role of miRNA polymorphisms in determining the risk of OPLL. Herein, we investigated the genetic association between four these SNPs in pre-miRNAs and cervical OPLL risk in the Korean population.

## Materials and Methods

### Study population

Subjects were recruited from the populations of South Korean provinces of Seoul and Gyeonggi-do between January 2014 and July 2015. This study was approved by Institutional Review Board of CHA Bundang Medical Center (IRB number: BD2014007). We selected 207 consecutive patients with cervical OPLL, who visited CHA Bundang Medical Center. Diagnoses were made by computed tomography (CT) and magnetic resonance imaging (MRI) examination by two independent experienced neurosurgeons. Dual-energy X-ray absorptiometry (DEXA) scan was performed on the lower spine and hips for measuring bone mineral density. All patients met the following eligibility criteria: 1) no metabolic diseases; such as diffuse idiopathic skeletal hyperostosis, pituitary diseases, and hyperparathyroidism; 2) no treatment interfering with bone metabolism or coagulation, including anticoagulants, oral contraceptives, hormones replacement therapy, glucocorticoids, calcium, or vitamin D; 3) no ankylosing spondylitis and spondylosis deformans; 4) normal bone mass (BMD: T-score, -1.0 through +1.0); 5) no prior stroke or ischemic heart disease. The ossified lesions were classified into four types (1) continuous type, which involves a long lesion extending over several vertebral bodies; (2) segmental type, which involves a few or several separate lesions behind the vertebral bodies; (3) mixed type, which is a combination of the first two, and (4) localized type, which involves a lesion at the level of intervertebral disc [38].

Clinical studies on OPLL revealed that patients with continuous and mixed type OPLL have a higher risk of progression of the ossification area and a worse prognosis, compared to patients with segmental and localized type [39]. There are genetic differences in the osteogenic differentiation potency between the OPLL continuous (continuous and mixed type) and segmental (segmental type plus localized type) groups [40]. Therefore, we categorized the four types of cervical OPLL into two groups: the OPLL segmental group (segmental and localized types) and the OPLL continuous group (continuous and mixed types).

We recruited 200 control subjects who underwent cervical CT due to persistent posterior neck pain after a rear-end motor vehicle collision. Control subjects had no neurological signs, including radiculopathy and/or myelopathy, and did not have a recent history of cerebrovascular disease or myocardial infarction. Cervical CT did not show any evidence of OPLL, cervical spinal stenosis, and spondylosis deformans. We utilized similar exclusion criteria as that was used in patient group, as described above. Demographic features and comorbidities such as hypertension, diabetes mellitus (DM), and cerebro-and cardiovascular diseases were investigated in all subjects. Hypertension was defined as systolic blood pressure was >140 mmHg, or a diastolic pressure >90 mmHg on more than one occasion, including those patients taking antihypertensive medications. DM was defined as a fasting plasma glucose level ≥126 mg/dL, including in patients taking insulin or oral hypoglycemic agent. Written consents were obtained for all the participants.

### Genetic analyses

DNA was extracted from leukocytes using the G-DEX^TM^ II Genomic DNA Extraction kit (iNtRON Biotechnology, Seongnam, South Korea), according to the manufacturer’s instructions. The polymerase chain reaction -restriction fragment length polymorphism (PCR-RFLP) was used to genotype the *miR-146a*C>G, *miR-149*T>C, *miR-196a2*T>C, and *miR*-*499*A>G SNPs as described previously [16–21]. Briefly, one microliter from each sample was used to amplify *miR-146a*, *miR-196a*, and *miR-499* genes. PCR primers were designed using PrimerQuest^TM^ (Integrated DNA Technologies, Coralville, IA, USA). The actual concentration of the DNA was 100ng/μl and all DNA samples were normalized to 100ng/μl for use in PCR. The primer sequences used for amplification were as follows: *miR-146a* C>G: forward 5′-CAT GGG TTG TGT CAG TGT CAG A**G**C T-3′ and reverse 5′-TGC CTT CTG TCT CCA GTC TTC CAA-3′; *miR-149* T>C: forward 5′-CTG GCT CCG TGT CTT CAC TC-3′ and reverse 5′-TGA GGC CCG AAA CAC CCG TA-3′; *miR-196a2* T>C: forward 5′-CCC CTT CCC TTC TCC TCC AGA TA-3′ and reverse 5′-CGA AAA CCG ACT GAT GTA ACT C**C**G-3′; and *miR*-*499*A>G: forward 5′-CAA AGT CTT CAC TTC CCT GCC A-3′ and reverse 5′-GAT GTT TAA CTC CTC TCC ACG TGA **T**C-3′ (Fig 1). The underlined bases represent mismatches with the complementary sequence. The *miR-146a*C>G, *miR-149*T>C, *miR-196a2*T>C, and *miR*-*499*A>G polymorphisms were detected by digesting the PCR products with *Sac*I, *Alu*I, *Msp*I, and *Bcl*I, respectively (New England BioLabs, Beverly, MA, USA). The reaction products (12 μl) were run on a 3.0% ethidium bromide-stained agarose gel and directly visualized under ultraviolet illumination. For each of the miRNA polymorphisms, approximately 10% of the PCR assays were randomly selected for a second PCR assay to validate the RFLP analysis, followed by DNA sequencing [16–21]. DNA sequencing was performed with an automatic sequencer (ABI3730x I DNA analyzer; Applied Biosystems, Foster City, CA, USA). The concordance of the quality control sample was 100%.

We analyzed gene-gene interaction among the four miRNAs loci using the multifactor dimensionality reduction (MDR) method (MDR software package, v.2.0, www.epistasis.org) [18, 20, 41–45]. The MDR consists of two main steps. First, the best combination of multifactors are selected. Second, the genotype combinations are divided into high- and low-risk groups [46]. Using MDR method, we constructed all possible allele combinations of the four SNPs. The HAPSTAT software (v.3.0, www.bios.unc.edu/~lin/hapstat/) was used to estimate the frequencies of allele combinations for the polymorphisms selected by MDR analysis with strong synergistic effects.

### Statistical analyses

Data analysis was performed using STATSDIRECT software version 2.4.4 (StatsDirect Ltd., Altrincham, UK) and GRAPHPAD PRISM 4.0 (GraphPad Software, Inc., San Diego, CA, USA). The odds ratio (OR) and confidence interval (CI) were calculated to estimate the relative risk of the four SNPs for cervical OPLL. Variableare presented as mean ± standard deviation compared using a Student *t* test for continuous variables and the Chi-square test for categorical variables between case abd control groups. Logistic regression analyses were to adjust possible confounders, including age, gender, hypertension and DM. A probability (*P*) value of 0.05 was considered to indicate statistical significance.

## Results

### Study population

The clinical characteristics of OPLL and control subjects are summarized in Table 1. There was no statistically significant difference for age and gender. Additionally, the prevalence of hypertension and DM did not differ between controls and patients with OPLL (Table 1). All OPLL patients underwent surgery during their admission due to severe myelopathy. In patients with an occupying rate (area occupied by OPLL/area of the total spinal canal) less than 50% and no more than three ossified segments, anterior approach (corpectomy and resection of OPLL) was chosen. Patients with massive OPLL with an occupying rate 50% or more and more than three levels were treated by posterior approach (laminectomy and lateral mass fusion, or laminoplasty) or combined anterior and posterior approach. One hundred and twenty six patients (60.9%) underwent a posterior-only approach, and 76 (36.7%) underwent an anterior-only approach and 5 (2.0%) underwent the combined approach. Of the 207 OPLL patients, 134 were part of the OPLL continuous group (continuous type; n = 62 and mixed type; n = 72) and 73 constituted the OPLL segmental group (segmental type; n = 38 plus localized type; n = 35).

**Table 1:** Baseline characteristics in patients and control subjects.

Characteristics	Control (%)	Case (%)	*P*
**Patient number**	200	207	
**Male (%)**	114 (57.0)	142 (68.6)	0.264
**Age (years, mean ± SD)**	53.58 ± 9.45	54.63 ± 9.70	0.268
**Hypertension**	81 (40.5)	89 (43.0)	0.611
**DM**	29 (14.5)	38 (18.4)	0.273

Values are the mean ± standard deviation or n (%) of participants. DM, diabetes mellitus. *P*-values are chi-square test for the categorical data, and the Student *t* test for the continuous data

### Genotype frequencies of miRNA polymorphisms

We investigated *miR-146a*C>G, *miR-149*T>C, *miR-196a2*T>C, and *miR*-*499*A>G polymorphisms (S1 Table). Table 2 shows their genotype distributions in patients with OPLL and control subjects. The four studied miRNA polymorphisms were in complete Hardy-Weinberg equilibrium. There were no significant difference in genotypic frequencies of *miR-146a*, *miR-149*, *miR-196a2*, and *miR*-*499* SNPs between OPLL patients and controls. We calculated the adjusted OR (AOR) from logistic regression analyses on age, gender, hypertension, and DM. There was no significant difference between groups even after AOR from logistic regression analyses (Table 2). A subgroup analysis showed a significantly increased risk of OPLL for subjects with the *miR*-*499*A>G polymorphism (AA+AG vs GG: AOR, 4.314; 95% CI, 1.088–26.293, *P* = 0.035) in the segmental OPLL subgroup (Table 3). However, we did not find any significant association between the control and patients groups in the continuous OPLL subgroup.

**Table 2:** AOR values of OPLL prevalence among four microRNAs genotypes in samples.

Characteristics	Controls (n = 200)	OPLL (n = 207)	COR (95% CI)	*P*	AOR (95% CI)^a^	*P*^b^
***miR-146a*C>G**						
CC	70 (35.0)	74 (35.7)	1.000 (reference)		1.000 (reference)	
CG	97 (48.5)	103 (49.8)	1.005 (0.654–1.542)	0.984	1.038 (0.673–1.601)	0.866
GG	33 (16.5)	30 (14.5)	0.860 (0.475–1.556)	0.618	0.808 (0.442–1.476)	0.487
Dominant (CC vs CG+GG)			0.968 (0.645–1.453)	0.875	0.976 (0.647–1.470)	0.906
Recessive (CC+CG vs GG)			0.858 (0.501–1.469)	0.576	0.801 (0.465–1.381)	0.424
HWE *P*	0.950	0.543				
***miR-149*T>C**						
TT	104 (52.0)	98 (47.3)	1.000 (reference)		1.000 (reference)	
TC	74 (37.0)	94 (45.4)	1.348 (0.894–2.033)	0.154	1.309 (0.862–1.989)	0.207
CC	22 (11.0)	15 (7.2)	0.724 (0.355–1.475)	0.373	0.652 (0.316–1.346)	0.247
Dominant (TT vs TC+CC)			1.205 (0.817–1.778)	0.348	1.160 (0.782–1.721)	0.460
Recessive (TT+TC vs CC)			0.632 (0.318–1.257)	0.191	0.589 (0.294–1.180)	0.136
HWE *P*	0.118	0.237				
***miR-196a2*T>C**						
TT	57 (28.5)	60 (29.0)	1.000 (reference)		1.000 (reference)	
TC	95 (47.5)	101 (48.8)	1.010 (0.639–1.597)	0.966	1.025 (0.645–1.630)	0.916
CC	48 (24.0)	46 (22.2)	0.910 (0.529–1.567)	0.735	0.861 (0.496–1.493)	0.594
Dominant (TT vs TC+CC)			0.977 (0.636–1.500)	0.914	0.968 (0.628–1.494)	0.884
Recessive (TT+TC vs CC)			0.905 (0.571–1.435)	0.671	0.849 (0.532–1.357)	0.495
HWE *P*	0.497	0.777				
***miR-449*A>G**						
AA	143 (71.5)	146 (70.5)	1.000 (reference)		1.000 (reference)	
AG	53 (26.5)	53 (25.6)	0.980 (0.628–1.529)	0.927	0.984 (0.628–1.542)	0.944
GG	4 (2.0)	8 (3.9)	1.959 (0.577–6.650)	0.281	2.064 (0.598–7.129)	0.252
Dominant (AA vs AG+GG)			1.048 (0.683–1.609)	0.830	1.057 (0.686–1.629)	0.801
Recessive (AA+AG vs GG)			1.970 (0.584–6.648)	0.275	2.049 (0.600–6.999)	0.252
HWE *P*	0.721	0.260				

^a^ Adjusted by age, gender, hypertension and diabetes mellitus.

^b^ False positive discovery rate-adjusted P-value.

OPLL: Ossification of posterior longitudinal ligament, COR: crude odds ratio, AOR: adjusted odds ratio

**Table 3:** AOR values of continuous OPLL subgroup and segmental OPLL subgroup prevalence among four microRNAs genotypes in samples.

Characteristics	Controls (n = 200)	Continuous +mixed (n = 135)	AOR^a^ (95% CI)	*P*^b^	Segmental +localized (n = 65)	AOR^a^ (95% CI)	*P*^b^
***miR-146a*C>G**							
CC	70 (35.0)	45 (33.3)	1.000 (reference)		23 (35.4)	1.000 (reference)	
CG	97 (48.5)	69 (51.1)	1.171 (0.714–1.921)	0.531	33 (50.8)	1.071 (0.576–1.990)	0.829
GG	33 (16.5)	21 (15.6)	0.893 (0.451–1.767)	0.745	9 (13.8)	0.866 (0.356–2.108)	0.752
Dominant (CC vs CG+GG)			1.108 (0.694–1.771)	0.667		1.029 (0.569–1.859)	0.925
Recessive (CC+CG vs GG)			0.850 (0.463–1.561)	0.601		0.811 (0.363–1.813)	0.609
***miR-149*T>C**							
TT	104 (52.0)	66 (48.9)	1.000 (reference)		28 (43.1)	1.000 (reference)	
TC	74 (37.0)	60 (44.4)	1.197 (0.749–1.914)	0.452	33 (50.8)	1.565 (0.864–2.832)	0.139
CC	22 (11.0)	9 (6.7)	0.551 (0.234–1.295)	0.172	4 (6.2)	0.588 (0.184–1.878)	0.370
Dominant (TT vs TC+CC)			1.048 (0.671–1.637)	0.836		1.342 (0.757–2.378)	0.315
Recessive (TT+TC vs CC)			0.509 (0.224–1.159)	0.108		0.514 (0.169–1.566)	0.242
***miR-196a2*T>C**							
TT	57 (28.5)	40 (29.6)	1.000 (reference)		18 (27.7)	1.000 (reference)	
TC	95 (47.5)	59 (43.7)	0.925 (0.544–1.572)	0.773	38 (58.5)	1.277 (0.665–2.451)	0.463
CC	48 (24.0)	36 (26.7)	0.999 (0.546–1.831)	0.998	9 (13.8)	0.612 (0.248–1.508)	0.286
Dominant (TT vs TC+CC)			0.954 (0.585–1.556)	0.850		1.061 (0.565–1.993)	0.853
Recessive (TT+TC vs CC)			1.073 (0.643–1.789)	0.789		0.497 (0.227–1.089)	0.081
***miR-449*A>G**							
AA	143 (71.5)	96 (71.1)	1.000 (reference)		45 (69.2)	1.000 (reference)	
AG	53 (26.5)	36 (26.7)	0.998 (0.603–1.651)	0.993	15 (23.1)	0.865 (0.442–1.693)	0.673
GG	4 (2.0)	3 (2.2)	1.189 (0.252–5.603)	0.827	5 (7.7)	4.298 (1.079–17.127)	0.039
Dominant (AA vs AG+GG)			1.011 (0.619–1.651)	0.965		1.090 (0.590–2.016)	0.783
Recessive (AA+AG vs GG)			1.196 (0.256–5.588)	0.820		4.314 (1.109–16.778)	0.035

^a^ Adjusted by age, gender, hypertension and diabetes mellitus.

^b^ False positive discovery rate-adjusted *P*-value.

OPLL: Ossification of posterior longitudinal ligament, COR: crude odds ratio, AOR: adjusted odds ratio

To explore the possible gene-gene interaction, we constructed possible allele combinations of *miR-146a*, *miR-149*, *miR-196a2*, and *miR-499* as described previously [18,20,41–46]. Interestingly, there was significant difference in several allele combination frequencies in patients with OPLL in comparison to control subjects (Table 4). When patients with OPLL were compared to control subjects, *miR-146a*C/*-149*T/*-196a2*T/*-499*G (AOR, 2.346, 95% CI, 1.084–5.075, *P* = 0.037), *miR-146a*G/*-149*T/*-196a2*T/*-499*A (AOR, 2.231, 95% CI, 1.345–3.699, *P* = 0.003), and *miR-146a*G/*-149*T/*-196a2*C/*-499*G (AOR, 37.040, 95% CI, 2.170–632.500, *P < 0*.*0001*) were significantly associated with an increased OPLL risk. In addition, *miR-146a*G/*-149*T/*-196a2*T/*-499*G (AOR, 0.044, 95% CI, 0.003–0.752, *P* = 0.001) and *miR-146a*G/*-149*C/*-196a2*C/*-499*G (AOR, 0.061, 95% CI, 0.004–1.057, *P* = 0.006) were significantly associated with a decreased OPLL risk (Table 4). We further compared the combined genotype distributions of *miR-146a*, *miR-149*, *miR-196a2*, *and miR*-*499* in patients with OPLL to controls (Table 5). The combined genotype analysis showed a significant association between miR-*149*TC/*-196a2*TT combined genotype and OPLL risk (AOR, 2.322, 95% CI, 1.043–5.171, *P* = 0.039).

**Table 4:** The haplotype analysis of the *miR-146a*G>C, *miR-149*T>C, *miR196a2*T>C, and *miR499*A>G polymorphisms between the control group and patients with OPLL.

Characteristics	Control (2n = 400)	OPLL (2n = 414)	OR (95% CI)	P*
*miR-146a*G>C/*miR-149*T>C/*miR-196a2*T>C/*miR-499*A>G				
C-T-T-A	87 (21.8)	68 (16.5)	1.000 (reference)	
C-T-T-G	12 (3.0)	22 (5.2)	2.346 (1.084–5.075)	0.037
C-T-C-A	63 (15.7)	76 (18.4)	1.543 (0.974–2.447)	0.080
C-T-C-G	11 (2.7)	5 (1.2)	0.582 (0.193–1.754)	0.430
C-C-T-A	26 (6.5)	27 (6.5)	1.329 (0.711–2.483)	0.426
C-C-T-G	5 (1.2)	9 (2.2)	0.168 (0.738–7.191)	0.168
C-C-C-A	28 (6.9)	34 (8.3)	1.554 (0.859–2.810)	0.176
C-C-C-G	6 (1.5)	10 (2.5)	2.132 (0.738–6.161)	0.191
G-T-T-A	39 (9.9)	68 (16.5)	2.231 (1.345–3.699)	0.003
G-T-T-G	14 (3.4)	0 (0.0)	0.044 (0.003–0.752)	0.001
G-T-C-A	56 (14.1)	37 (8.9)	0.845 (0.501–1.426)	0.596
G-T-C-G	0 (0.0)	14 (3.5)	37.040 (2.170–632.500)	<0.0001
G-C-T-A	22 (5.5)	18 (4.3)	1.047 (0.520–2.106)	1.000
G-C-T-G	4 (1.0)	9 (2.2)	2.879 (0.850–9.751)	0.090
G-C-C-A	17 (4.4)	17 (4.0)	1.279 (0.608–2.691)	0.570
G-C-C-G	10 (2.6)	0 (0.0)	0.061 (0.004–1.057)	0.006

Haplotypes of frequencies <5% and not significant were excluded.

* *P*-values were calculated by Fisher's exact test.

OPLL: Ossification of posterior longitudinal ligament, OR: odds ratio

**Table 5:** Comparison microRNAs combined genotype between the controls and patients.

Characteristics	Control (n = 200)	OPLL (n = 207)	AOR (95% CI)	*P*
*miR-146a/miR-149*				
CC/TT	40 (20.0)	38 (18.4)	1.000 (reference)	
CC/TC	23 (11.5)	27 (13.0)	1.285 (0.610–2.703)	0.510
CG/TT	51 (25.5)	45 (21.7)	0.971 (0.527–1.791)	0.926
CG/TC	33 (16.5)	52 (25.1)	1.791 (0.941–3.408)	0.076
CG/CC	13 (6.5)	6 (2.9)	0.396 (0.131–1.195)	0.100
GG/TT	13 (6.5)	15 (7.2)	1.232 (0.509–2.986)	0.644
GG/TC	18 (9.0)	15 (7.2)	0.768 (0.331–1.784)	0.540
*miR-146a/miR-196a2*				
CC/TT	25 (12.5)	19 (9.2)	1.000 (reference)	
CC/TC	30 (15.0)	38 (18.4)	2.125 (0.924–4.888)	0.076
CC/CC	15 (7.5)	17 (8.2)	1.312 (0.494–3.485)	0.586
CG/TT	22 (11.0)	28 (13.5)	2.181 (0.878–5.419)	0.093
CG/TC	51 (25.5)	51 (24.6)	1.459 (0.702–3.032)	0.312
CG/CC	24 (12.0)	24 (11.6)	1.395 (0.604–3.223)	0.436
GG/TT	10 (5.0)	13 (6.3)	1.902 (0.661–5.472)	0.233
GG/TC	14 (7.0)	12 (5.8)	1.176 (0.422–3.276)	0.756
*miR-146a/miR-499*				
CC/AA	51 (25.5)	51 (24.6)	1.000 (reference)	
CC/AG	18 (9.0)	20 (9.7)	1.145 (0.530–2.474)	0.730
CG/AA	69 (34.5)	72 (34.8)	1.080 (0.643–1.815)	0.771
CG/AG	26 (13.0)	26 (12.6)	1.101 (0.554–2.186)	0.784
GG/AA	23 (11.5)	23 (11.1)	0.957 (0.472–1.940)	0.902
*miR-149/miR-196a2*				
TT/TT	34 (17.0)	25 (12.1)	1.000 (reference)	
TT/TC	46 (23.0)	57 (27.5)	1.763 (0.904–3.436)	0.096
TT/CC	24 (12.0)	16 (7.7)	0.869 (0.379–1.990)	0.739
TC/TT	19 (9.5)	30 (14.5)	2.322 (1.043–5.171)	0.039
TC/TC	36 (18.0)	38 (18.4)	1.398 (0.695–2.812)	0.348
TC/CC	19 (9.5)	26 (12.6)	1.772 (0.795–3.950)	0.162
*miR-149/miR-499*				
TT/AA	77 (38.5)	74 (35.7)	1.000 (reference)	
TT/AG	25 (12.5)	21 (10.1)	0.854 (0.434–1.680)	0.648
TC/AA	52 (26.0)	62 (30.0)	1.178 (0.715–1.940)	0.521
TC/AG	22 (11.0)	27 (13.0)	1.289 (0.669–2.484)	0.448
*miR-196a2/miR-499*				
TT/AA	39 (19.5)	40 (19.3)	1.000 (reference)	
TT/AG	17 (8.5)	17 (8.2)	0.948 (0.415–2.169)	0.900
TC/AA	70 (35.0)	74 (35.7)	1.016 (0.582–1.775)	0.956
TC/AG	23 (11.5)	23 (11.1)	1.058 (0.501–2.238)	0.882
CC/AA	34 (17.0)	32 (15.5)	0.825 (0.421–1.616)	0.575
CC/AG	13 (6.5)	13 (6.3)	0.863 (0.348–2.143)	0.751

The frequencies < 5% and not significant were excluded. OPLL: Ossification of posterior longitudinal ligament, AOR: adjusted odds ratio.

## Discussion

In this case-control study of 407 total Korean patients, we found that the *miR-499*GG genotype was associated with an increased risk of OPLL in the segmental OPLL subgroup. In addition, some allele combinations (*miR-146a*C/*-149*T/*-196a2*T/*-499*G, *miR-146a*G/*-149*T/*-196a2*T/*-499*A, and *miR-146a*G/*-149*T/*-196a2*C/-*499*G) were associated with an increased OPLL risk, whereas *miR-146a*G/*-149*T/*-196a2*T/-*499*G and *miR-146a*G/*-149*C/*-196a2*C/-*499*G had a protective role in OPLL pathogenesis. To the best of our knowledge, this is the first study to examine the role of miRNA polymorphisms in the pathogenesis of OPLL.

The exact mechanisms of OPLL initiation and promotion remain unclear. The endochondral ossification process in OPLL is associated with degenerative changes in elastic fibers and cartilage formation as well as with changes in vascular endothelial growth factor (VEGF)-positive metaplastic chondrocytes in the ossification front [47]. Additionally, OPLL results from abnormal osteogenic differentiation of the ligaments, secondary to inflammation, or systemic dysfunction of bone metabolism [2,3,5]. Thus, the susceptibility to OPLL may be mediated by changes in the genes encoding proteins that are related to osteogenesis, inflammation, and bone metabolism [1,3–11]. Based on the classification of OPLL, the OPLL continuous (continuous and mixed type) subgroup can present a higher risk of aggravating myelopathy compared to the OPLL segmental subgroup (segmental and localized type) [39]. Interestingly, there are genetic differences in the osteogenic differentiation potential between the OPLL continuous and OPLL segmental subgroups [40].

miRNAs can influence osteogenic differentiation and affect bone metabolism or the bone healing process. Accumulating evidence recently indicated a vital role of miRNAs in the development of various bone diseases [48–53]. miRNAs appears to regulate posttranscriptional gene expression by binding to the 3′-UTRs of the target mRNAs [11] and SNPs located in the gene 3′-UTRs of mRNAs indeed significantly contribute to the variation in term of expression [54]. Our study demonstrated that 4 well-known SNPs in pre-miRNA sequences (*miR-146a*, *miR-149*, *miR-196*a2, and *miR-499*) are associated with various diseases [16–21]. As shown in Fig 1, SNPs in *miR-146a*, *miR-196a2*, *and miR*-*499* are all located in their corresponding 3p mature miRNA regions [14, 22–26], and they may impact miRNA-target interaction leading to influence mature miRNA expression and target gene expression [14, 22–26].

Understanding the potential roles of the four miRNAs is important to elucidate OPLL etiologies. However, we could not demonstrate *in vitro* functionalities of *miR-146a*, *-149*, *-196a2*, *-499* genetic variations in the present study. The several studies indicated a potential association of four miRNAs with the pathways of osteogenesis and inflammation. *miR-146a* may have a role in the regulation of osteogenesis by attenuating SMAD2 and SMAD3 function in transforming growth factor-β (TGF-β) signaling [29]. Additionally, *miR-146a* may be involved in inflammation by suppressing the effects of inflammatory factors and human chondrocyte apoptosis by increasing the levels of VEGF and damaging TGF-β signaling [30]. Tumor necrosis factor (TNF)-α enhances osteogenic differentiation of human adipose tissue-derived mesenchymal stem cells through nuclear factor κB (NF-κB) activation. *miR-146a* overexpression could suppress osteogenic differentiation and block NF-κB activation induced by TNF-α and toll-like receptor ligands [28]. A defect in the *miR-146a-*mediated negative loop may provide the environment for sustained activation of NF-κB and its targets to promote cells toward inflammation, apoptosis, and subsequent abnormalities [35,55]. MTHFR may be a target gene of *miR-149* [29]. Numerous studies have reported an association between the *MTHFR* polymorphism and reduced bone mineral density, but results have been inconsistent [53,56]. A recent systematic meta-analysis showed that the *MTHFR* 677C>T polymorphism is associated with reduced bone mineral density in the lumbar spine and femoral neck in Caucasians, postmenopausal women, and men, and total body bone mineral density in women [56]. ANXA1 (annexin A1), a possible target gene of *miR-196a2*, regulates bone marrow-derived mesenchymal stem cell proliferation and osteogenic differentiation [32,57]. *miR-499* may be involved in autoimmune and inflammatory diseases. The possible targets of *miR-499* include IL-17Rβ, IL-23α, IL-2R, IL-6, IL-2, and IL-18R. IL-6 could activate the production of CRP and fibrinogen through the liver [34,35,58]. We hypothesized that chronic inflammation and abnormal osteogenic differentiation could play a role in the initiation and progression of OPLL and analyzed SNPs in 4 miRNAs (*miR-146*a, *miR-149*, *miR-196a2*, and *miR-499*), which are known to be involved in inflammation and chondrogenic differentiation. In terms of a target of *miR-499*, SOX6 is one of targets of miR-499 [59], and SOX6 has been reported to play an important role in the endochondral ossification [60]. However, our data do not prove a direct interaction of the target gene with the 4 miRNAs and our results should be interpreted with caution.

There are several limitations to this study. (1) The study population comprised only Korean individuals and we cannot generalize our finding to other ethnic populations. Thus, present findings need to be replicated and validated in other ethnic groups because SNPs and haplotype structures can vary among different ethnic groups. (2) The relatively small sample size of individual with OPLL subtype were used in current hospital-based case-control study. (3) We could not conclusively rule out some other potential confounders such as exposure to different environmental factors (such as smoking) and additional genetic factors. (4) We could not prove that SNPs in the pre-miRNA region of *miR-146*a/*-149/-196a2/-499* can actually affect miRNA binding to target genes involved in OPLL in vitro. In the future, large, community-based random sampling and in vitro study for validation of miRNA-target interaction will be required to run replication studies in order to resolve partially these limitations.

In conclusion, the *miR-499*GG genotype is associated with an increased risk of OPLL in the segmental OPLL subgroup and that the *miR-146a/-149/-196a2/*-*499* allele combinations may be a genetic risk factor for cervical OPLL in the Korean populations. This study marks the first report of an association between cervical OPLL and miRNA polymorphisms (*miR-146a*C>G, *-149*T>C, *-196a2*T>C, and -*499*A>G) in the Korean population. Multiple variants with small effects and complex network underlie the pathogenesis of OPLL. Thus, additional studies on other racial and ethnic populations regarding the biological functions of miRNAs in OPLL are required.

## Supporting Information

S1 Table Raw data of four microRNAs genotypes in all the subjects. (PDF)
