# Supplementary material for: Association of miR-146a, miR-149, miR-196a2, and miR-499 Polymorphisms with Ossification of the Posterior Longitudinal Ligament of the Cervical Spine
Source: PLoS One. 2016 Jul 25;11(7):e0159756. doi: 10.1371/journal.pone.0159756 (PMC4959720; doi:10.1371/journal.pone.0159756)
Supplement: S1 Table — (PDF) [file pone.0159756.s001.pdf]

Outcome	Number	Sex	Age	miR-146a rs2910164C>G	miR-149 rs2292832T>C	miR-196a2 rs11614913C>T	miR-499 rs3746444A>G
0	1	1	59	CC	CT	CC	AA
0	2	1	59	GC	TT	CT	AA
0	3	1	64	GC	TT	CT	AA
0	5	1	67	CC	TT	TT	AA
0	7	2	61	GG	TT	CC	AA
0	8	1	46	CC	CT	TT	AA
0	11	1	68	CC	TT	CT	AA
0	12	2	60	GG	TT	CT	AA
0	16	1	51	CC	CC	TT	AA
0	18	1	68	GC	TT	CC	AA
0	19	2	57	GG	CT	TT	AA
0	20	1	55	GC	TT	CT	AA
0	23	1	59	GC	TT	CT	AA
0	24	1	52	CC	TT	CT	AA
0	26	1	69	GC	CC	CT	AA
0	29	2	53	GG	TT	CT	AG
0	34	1	63	CC	CT	CC	AA
0	39	2	43	GC	CT	TT	AA
0	40	1	63	GC	TT	CT	AG
0	42	1	69	GC	TT	CC	AA
0	43	1	64	GG	CT	CT	AA
0	44	1	62	GC	CT	CC	AG
0	46	2	40	GC	TT	TT	AA
0	47	2	44	GC	TT	CC	AA
0	49	2	48	GC	TT	CC	AA
0	51	1	67	GG	CC	CC	AA
0	53	1	64	CC	TT	TT	AA
0	54	1	51	GG	CT	CT	AA
0	56	1	49	GG	CT	TT	AA
0	59	1	54	GG	CT	CT	AA
0	60	2	47	CC	TT	CT	AA
0	61	1	50	GC	TT	CC	AA
0	64	1	62	CC	TT	TT	AG
0	69	1	56	GC	CT	CC	AA
0	70	1	32	GC	TT	TT	AG
0	71	1	59	GG	CT	TT	AA
0	72	1	60	CC	CT	CC	AA
0	74	2	47	GC	CT	TT	AA
0	75	2	49	CC	CC	CT	AA
0	77	1	48	GG	CT	TT	AA
0	79	2	60	GG	TT	TT	AA

0	83	2	44	CC	TT	CC	GG
0	84	1	61	GC	CC	CC	AG
0	89	1	60	CC	CT	CT	AA
0	92	1	43	GG	CC	CT	GG
0	94	2	55	GC	CC	CT	AA
0	95	1	22	GC	CT	CT	AG
0	96	1	39	GG	TT	CC	AA
0	100	1	39	GC	TT	TT	AA
0	102	1	58	CC	TT	TT	AG
0	104	2	59	GC	TT	CT	GG
0	108	1	50	CC	CT	TT	AA
0	113	2	46	GC	CT	CT	AA
0	114	1	58	GC	TT	CT	AA
0	115	1	47	GC	TT	CT	AA
0	117	1	61	GG	CT	TT	AG
0	120	1	66	GC	TT	CT	AA
0	121	2	47	GG	TT	CT	AA
0	122	2	43	CC	CC	CC	AG
0	125	1	48	GG	TT	TT	AA
0	127	1	57	GC	TT	TT	AG
0	131	1	65	GG	CT	TT	AA
0	133	2	44	CC	TT	CT	AA
0	135	1	67	CC	TT	CC	AA
0	136	1	65	GC	CT	CT	AA
0	137	1	63	GC	CT	CC	AG
0	141	2	44	GC	TT	CT	AA
0	142	2	46	GC	TT	TT	AG
0	143	1	34	CC	TT	TT	AG
0	145	1	61	CC	TT	CT	AG
0	148	2	43	CC	CT	TT	AG
0	149	1	44	GG	TT	TT	AG
0	150	2	54	CC	TT	CT	AA
0	153	1	42	GC	CT	CT	AA
0	155	2	50	GC	CT	TT	AA
0	156	1	67	GC	TT	TT	AA
0	157	2	61	GG	CT	CT	AA
0	158	2	25	GC	TT	CT	AA
0	161	1	53	CC	TT	CT	AA
0	162	1	32	CC	CT	CT	AA
0	168	2	60	GC	CT	CT	AA
0	170	2	33	CC	TT	CT	AA
0	171	1	45	CC	CT	TT	AG
0	176	1	39	GC	CT	CT	AA

0	177	1	39	GC	CT	CC	AA
0	178	2	58	GG	TT	CT	AG
0	179	2	62	GC	TT	CC	AA
0	181	1	62	GC	CC	CC	AG
0	182	1	43	CC	TT	CT	AA
0	183	2	48	CC	TT	CT	AA
0	186	1	64	GG	TT	CC	AA
0	190	2	46	GG	TT	CT	AG
0	192	1	55	CC	CT	CT	AA
0	193	1	60	CC	TT	TT	AA
0	196	1	60	GC	CC	CT	AA
0	199	2	52	CC	TT	TT	AA
0	201	2	58	CC	TT	CC	AA
0	202	1	60	GC	CC	CT	AG
0	203	1	62	CC	TT	TT	AG
0	1	1	56	GG	CT	CT	AA
0	2	1	59	GG	TT	CT	AA
0	3	1	67	CC	CC	CT	AA
0	9	2	42	GC	TT	TT	AG
0	15	2	39	CC	TT	TT	AA
0	16	2	60	CC	CC	CC	AA
0	24	1	59	GC	TT	TT	AG
0	29	2	52	GC	CT	CT	AA
0	31	2	50	GC	TT	CT	AA
0	32	1	44	CC	CT	TT	AG
0	37	1	62	GC	CT	CT	AA
0	38	2	44	GC	TT	CC	AA
0	40	1	68	GC	CT	CC	AA
0	41	2	48	CC	TT	CT	AG
0	44	1	50	CC	CT	CT	AG
0	49	2	47	GG	CT	CC	AA
0	50	2	49	GC	TT	TT	AA
0	53	2	58	CC	TT	CT	AA
0	57	1	63	CC	CT	CT	AA
0	64	2	53	GC	CT	CC	AA
0	67	1	54	GC	CT	CT	AG
0	68	1	57	GC	TT	CC	AA
0	69	1	55	GC	CT	CT	AA
0	70	1	59	GC	CT	CC	AG
0	71	2	50	GC	TT	CT	AA
0	73	1	42	GC	CT	CT	AG
0	78	2	62	GC	CT	CT	AA
0	80	1	55	GC	TT	CT	AA

0	81	2	46	CC	TT	CC	AA
0	82	1	50	GC	TT	CC	AG
0	85	1	45	GC	CT	CT	AG
0	88	1	41	CC	CT	TT	AA
0	89	1	62	GC	TT	TT	AA
0	90	2	44	GC	CT	CT	AA
0	91	1	51	CC	CT	CT	AA
0	93	1	43	CC	TT	CT	AA
0	94	1	49	GC	TT	CC	AA
0	96	2	59	GC	CT	CC	AA
0	98	1	59	GC	CC	TT	GG
0	99	2	62	GC	TT	CT	AA
0	104	2	53	GC	TT	CT	AG
0	106	2	59	CC	CT	CT	AG
0	108	2	62	CC	TT	CC	AG
0	112	1	52	CC	TT	CC	AA
0	113	2	61	GC	TT	CT	AA
0	114	2	50	CC	CT	CT	AG
0	115	1	63	CC	CT	CC	AG
0	118	1	66	GC	CC	CT	AA
0	124	2	62	GG	CT	TT	AG
0	125	1	64	GG	CT	CC	AA
0	128	1	47	CC	CC	TT	AA
0	129	1	39	GC	CT	CC	AA
0	130	2	62	GC	CT	CT	AA
0	133	1	50	CC	TT	CC	AA
0	134	2	57	GC	CT	CC	AG
0	136	1	50	GG	CT	CC	AG
0	140	2	47	GC	CT	TT	AA
0	142	2	49	GC	TT	TT	AA
0	143	1	66	CC	TT	CC	AA
0	147	1	61	GC	TT	CT	AA
0	151	2	49	CC	TT	CT	AA
0	154	2	60	GC	CT	CT	AA
0	156	2	46	GC	TT	TT	AA
0	159	1	38	CC	CC	CT	AG
0	160	1	63	CC	TT	CT	AA
0	161	2	55	GC	CT	CT	AG
0	162	2	51	CC	CT	CT	AA
0	164	2	56	CC	TT	TT	AA
0	168	2	53	GC	TT	CT	AG
0	169	2	54	GC	TT	CC	AG
0	177	2	40	CC	TT	CT	AA

0	180	2	50	CC	CT	CT	AA
0	188	1	68	GG	TT	CC	AA
0	192	2	55	CC	TT	CT	AG
0	196	1	57	GC	TT	TT	AG
0	197	2	58	GC	TT	TT	AA
0	200	2	44	CC	TT	TT	AA
0	202	1	64	GC	CC	CT	AA
0	203	2	56	CC	CT	TT	AA
0	204	1	61	GG	CT	CC	AG
0	207	2	58	GC	TT	CT	AA
0	208	1	66	GC	CC	TT	AA
0	213	2	41	GC	TT	CT	AA
0	225	2	54	GC	TT	TT	AA
0	226	1	34	CC	CT	TT	AA
0	227	1	49	CC	TT	TT	AA
0	230	1	50	GC	CC	CT	AA
0	231	2	47	GC	TT	CT	AG
0	232	2	60	GC	TT	CC	AA
0	237	1	55	GC	TT	CT	AA
0	239	2	56	GC	TT	TT	AA
0	240	1	68	CC	TT	TT	AA
0	241	1	47	CC	CT	CC	AA
0	242	2	62	GC	CC	CT	AA
0	246	2	68	GG	CT	CT	AA
0	247	2	60	GC	CT	CT	AA
0	248	2	27	CC	TT	TT	AA
0	251	1	69	CC	TT	TT	AG
0	252	1	49	GC	CT	CT	AG
0	255	2	46	GC	CC	CT	AG
0	256	1	69	GG	CT	CT	AG
1	1	1	60	GG	CT	CC	AG
1	2	2	44	GC	TT	CT	AA
1	3	1	55	CC	TT	TT	AA
1	4	2	55	GC	TT	CC	AG
1	5	1	75	GC	TT	CT	AA
1	6	2	67	GC	TT	TT	AA
1	7	2	54	GC	CT	CT	AA
1	8	1	62	GC	TT	CT	AA
1	9	1	61	GC	TT	CT	AA
1	10	2	56	CC	TT	CT	AA
1	11	2	62	GC	CT	CT	AA
1	12	1	58	GC	TT	CT	AA
1	13	1	52	GC	TT	CT	AA

1	14	1	54	GC	TT	CT	AG
1	15	1	55	GC	TT	TT	AA
1	16	1	57	GG	TT	TT	AA
1	17	1	41	GC	CT	CC	AA
1	18	1	47	GG	TT	CC	AA
1	19	1	58	GG	TT	TT	AA
1	20	1	57	CC	CT	CT	AG
1	21	1	41	CC	CT	CT	AA
1	22	2	49	GC	CT	TT	AG
1	23	1	52	GC	CT	CC	AA
1	24	1	58	CC	TT	TT	AA
1	25	1	55	GG	CT	TT	AA
1	26	2	46	GC	CT	CT	AA
1	27	2	47	GC	CT	CT	AG
1	28	2	46	CC	CT	CT	AG
1	29	1	46	GC	TT	TT	AA
1	30	2	45	GC	TT	CT	GG
1	31	2	51	GC	CT	TT	AG
1	32	1	32	CC	TT	CT	AG
1	33	1	55	CC	TT	CC	AA
1	34	1	53	GC	TT	CC	AA
1	35	1	69	GC	CT	CT	AA
1	36	2	54	GG	TT	TT	AA
1	37	2	39	GC	TT	CC	AG
1	38	1	41	CC	TT	CT	AA
1	39	1	57	GC	CT	CT	GG
1	40	2	52	GC	TT	TT	AA
1	41	2	53	CC	TT	CT	AA
1	42	1	55	CC	TT	CT	AG
1	43	1	67	GC	TT	CT	GG
1	44	1	44	GC	CT	CT	AA
1	45	2	60	CC	TT	CT	AA
1	46	2	48	GC	TT	TT	AA
1	47	2	56	CC	TT	CT	AA
1	48	1	46	CC	CT	CC	AG
1	49	1	39	CC	CC	CT	AA
1	50	1	61	GC	TT	CT	AG
1	51	2	52	CC	TT	TT	AA
1	52	2	41	GC	TT	CC	AA
1	53	1	64	CC	TT	CC	AA
1	54	1	55	CC	TT	CT	AA
1	55	1	65	GC	TT	CT	AA
1	56	2	36	GC	TT	CT	AA

1	57	1	54	GC	TT	CT	AA
1	58	1	31	GC	CT	CT	AA
1	59	2	53	GC	CT	CC	AA
1	60	1	58	CC	CT	TT	AA
1	61	1	38	GC	TT	TT	AA
1	62	2	62	GC	TT	TT	AA
1	63	1	66	GC	CT	TT	AA
1	64	2	42	CC	CT	TT	GG
1	65	2	54	CC	CT	CT	AA
1	66	2	53	GC	CT	CT	AG
1	67	1	48	GG	CT	CT	AA
1	68	1	47	GC	CT	TT	AA
1	69	1	55	GC	TT	CT	AA
1	70	1	62	GG	CT	TT	AG
1	71	1	46	GG	CT	CT	AA
1	72	1	54	GC	CT	TT	AA
1	73	1	49	CC	CC	CT	AG
1	74	2	51	GC	CT	CC	AA
1	75	1	54	GC	CT	CT	AA
1	76	1	31	CC	TT	CT	AG
1	77	1	58	GC	TT	CT	AA
1	78	1	57	GC	CC	CC	AA
1	79	1	50	GC	CT	CT	AA
1	80	1	61	GC	CC	TT	AG
1	81	1	56	CC	TT	CT	AA
1	82	1	40	CC	TT	CT	AA
1	83	1	43	CC	CC	TT	AA
1	84	2	67	GC	TT	TT	AG
1	85	2	46	GG	TT	CT	AA
1	86	1	53	CC	CT	CC	AA
1	87	1	53	GC	TT	CT	AA
1	88	1	62	GC	CT	TT	AG
1	89	2	47	GC	TT	TT	AA
1	90	1	63	GC	CC	TT	AA
1	91	1	51	GG	CT	TT	AA
1	92	1	54	GG	TT	CT	AG
1	93	1	66	CC	CC	CC	AA
1	94	1	65	GG	CT	CT	AA
1	95	1	67	CC	TT	TT	GG
1	96	2	47	GC	CC	CC	AA
1	97	1	60	CC	TT	CT	AG
1	98	1	37	GC	CT	CT	AA
1	99	1	53	GC	TT	CT	AA

1	100	1	46	GC	TT	CC	AA
1	101	1	52	GC	CT	CC	GG
1	102	1	51	CC	TT	CC	AA
1	103	1	66	CC	CT	TT	AA
1	104	1	66	GC	CT	CT	AA
1	105	1	52	CC	TT	CT	AA
1	106	1	51	GC	CT	CT	AA
1	107	1	57	GG	TT	CC	AA
1	108	2	52	GC	CT	CC	AG
1	109	1	62	GC	TT	CC	AA
1	110	1	50	GC	CT	CT	AG
1	111	2	46	GC	CT	CT	AG
1	112	1	69	CC	TT	TT	AA
1	113	2	41	GG	CT	TT	AA
1	114	1	56	GC	TT	CT	AA
1	115	1	49	GG	TT	CT	AA
1	116	1	68	CC	CT	TT	AA
1	117	1	38	GC	CT	CT	AA
1	118	1	61	CC	TT	CT	AA
1	119	1	47	GG	TT	TT	AA
1	120	2	69	GC	TT	CT	AG
1	121	1	57	GC	TT	CC	AG
1	122	1	63	CC	CT	CC	AA
1	123	1	43	GG	CT	CT	AG
1	124	1	51	GC	CT	TT	AA
1	125	1	66	GC	TT	CT	AA
1	126	1	58	GC	CT	CT	AA
1	127	1	59	CC	CT	CC	AA
1	128	2	45	GC	CT	TT	AA
1	129	1	49	CC	CT	CC	AA
1	130	2	63	GC	CT	CT	AA
1	131	2	62	CC	TT	CT	AA
1	132	1	55	CC	CT	CT	AA
1	133	2	68	CC	CT	CC	AG
1	134	1	68	GC	CT	TT	AG
1	135	1	50	GG	TT	CT	AA
1	136	1	49	GC	TT	CT	AG
1	137	2	44	CC	CT	CC	AA
1	138	2	54	CC	CC	CT	AA
1	139	1	52	GG	TT	CC	AA
1	140	1	42	GC	CC	CC	AA
1	141	1	53	CC	CT	TT	AA
1	142	1	51	GG	CT	TT	AA

1	143	2	47	GC	TT	CT	AA
1	144	1	51	CC	CT	CT	AG
1	145	2	65	GG	TT	CT	AG
1	146	1	52	CC	TT	TT	AG
1	147	2	48	GC	CT	CT	AA
1	148	1	54	CC	CT	CT	AA
1	149	2	66	GG	TT	CT	AA
1	150	1	36	GC	CT	TT	AG
1	151	1	63	GC	TT	CT	AA
1	152	2	58	GC	CT	TT	GG
1	153	1	67	GG	CT	TT	AG
1	154	1	46	GC	TT	TT	AG
1	155	2	69	CC	CT	CC	AG
1	156	1	53	GC	TT	CC	AA
1	157	1	58	CC	TT	TT	AA
1	158	1	63	GG	TT	CT	AA
1	159	2	54	CC	TT	CT	AA
1	160	1	62	GC	CT	CC	AA
1	161	1	35	GC	CT	CT	AA
1	162	1	51	CC	CC	TT	AA
1	163	1	42	GG	CT	TT	AG
1	164	2	70	GC	CT	TT	AA
1	165	2	47	CC	CC	CT	AG
1	166	1	59	CC	CT	CC	AG
1	167	1	53	CC	CT	CT	GG
1	168	1	45	GC	TT	TT	AG
1	169	1	75	CC	TT	CT	AA
1	170	2	52	GC	TT	CT	AA
1	171	1	60	GG	TT	TT	AA
1	172	1	48	GC	CT	CC	AG
1	173	1	59	GC	TT	CT	AA
1	174	2	55	CC	TT	CT	AA
1	175	1	45	GC	CT	CT	AA
1	176	2	42	GG	CT	TT	AA
1	177	1	51	GC	CT	CC	AG
1	178	1	61	GG	CT	CC	AA
1	179	2	49	GC	CT	TT	AG
1	180	1	66	CC	TT	TT	AG
1	181	2	56	GC	CT	TT	AG
1	182	1	52	GC	TT	CT	AA
1	183	2	66	CC	TT	CT	AA
1	184	1	54	GC	CT	CC	AA
1	185	1	51	CC	CT	CC	AA

1	186	1	84	CC	CT	TT	AA
1	187	1	41	GC	CT	CT	AA
1	188	2	72	CC	CT	CC	AA
1	189	1	69	CC	TT	CC	AG
1	190	1	70	GG	CT	CT	AA
1	191	1	57	CC	TT	CT	AA
1	192	1	67	GC	CC	CT	AA
1	193	1	65	GC	CT	CC	AA
1	194	2	72	CC	CT	TT	AA
1	195	1	49	CC	CT	CT	AA
1	196	1	60	GC	CT	CC	AG
1	197	1	65	CC	TT	CT	AG
1	198	1	54	GC	CT	CC	AA
1	199	1	41	GC	CT	TT	AA
1	200	1	46	CC	TT	CT	AG
1	201	2	78	CC	TT	TT	AA
1	202	2	58	CC	TT	CT	AA
1	203	2	67	CC	CC	TT	AG
1	204	2	76	CC	CC	CT	AG
1	205	1	42	GC	CT	CT	AA
1	206	2	78	CC	TT	CC	AA
1	207	2	67	CC	TT	CT	AA
